# Proteomic Analysis of Microorganisms

**DOI:** 10.3390/ijms23084329

**Published:** 2022-04-14

**Authors:** Seung Il Kim

**Affiliations:** 1 Research Center for Bioconvergence Analysis, Korea Basic Science Institute (KBSI), Ochang 28119, Korea; ksi@kbsi.re.kr; 2 Bio-Analytical Science Division, University of Science and Technology, Daejeon 34113, Korea

At the early stage of the development of proteomic technologies, *Escherichia coli* or *Saccharomyces cerevisiae* were used as model microorganisms for high-throughput identification technologies, such as shotgun proteomics or 2D gel electrophoresis-based LC-MS/MS analysis. The merit of these microorganisms is the small size of their genome, which was elucidated in the early genome era. Subsequently, various environmental and clinical microorganisms have been applied in proteomic studies to understand their peculiar physiological and biochemical traits. However, cutting-edge omics technologies, such as NGS and transcriptomics, have been considered more useful and competitive techniques for bacterial studies of pure-cultured single microorganisms or bacterial communities (microbiomes) originating from various ecological niches. However, proteomics is still a useful technology in specific research fields as it can supplement the limits of genomic analysis of microorganisms. This Special Issue aims to collect articles showing the recent application of proteomic technologies for bacterial studies. Seven research papers and one review paper contributed valuable research results and provided insights into recent bacterial studies. They were categorized into five groups according to the purpose of the study.

The subcellular location of proteins can be predicted using various tools (SingalP, PSORTb, TMHMM, etc.) with relatively high accuracy. However, this prediction should be confirmed experimentally. Therefore, the verification of secreted or extracellular proteins is an important part of bacterial proteomics. In this Special Issue, two research papers focused on exoproteomes and extracellular vesicles (EVs). Moreira et al. [1] characterized EVs of the parasitic euglenoid *Trypanosoma cruzi*. Two stages (infective and non-infective) of *T. cruzi* produce different EVs, which show marked differential expression of the trans-sialidase protein. The authors explained that these proteins were related to cell adhesion. Based on the biophysical and biochemical comparative analysis of EVs, the authors suggested remarkable surface remodeling throughout of the *T. cruzi* life cycle. Savinova et al. [2] focused on the antagonistic potential of two probiotic lactic acid bacteria (LAB) against MDR *Klebsiella pneumoniae* under co-culture conditions and used proteomic tools as to screen for major role prayers. Proteomic analysis of secreted proteins of *L. reuteri* LR1 and *L. rhamnosus* F revealed many classically or non-classically secreted proteins and suggested cell wall-degrading enzymes and cell wall hydrolases as key enzymes for antagonistic interaction.

PTMs are important in diverse cellular functions but cannot be elucidated by genomic analysis. Therefore, novel PTM analysis methods are still under development in the proteomic field.

Ma et al. [3] performed a comprehensive quantitative phosphoproteomic profile analysis of coccidiosis-causing *E. tenella*. From the four life cycles, 198–456 differentially expressed phosphoproteins were identified. These proteins are involved in carbohydrate metabolism, cytoskeleton organization, and calcium ion transport. These findings shed light on the key role of phosphorous or dephosphorylation in the life cycle of *E. tenella*. Cysteine profiling is an important part of redox proteomic analysis. Hamitouche et al. [4] performed LC- MS/MS analysis to detect significant changes in the protein abundance and thiol status of cysteine-containing proteins in *Bacillus cereus* during aerobic exponential growth. Their results provide fundamental data for understanding the response mechanism of *Bacillus cereus* to deal with endogenous oxidative stress.

Lee and Kim [5] introduced clinical proteomics using human body fluids for the screening of diagnostic protein markers for infectious diseases, and summarized recent proteomic studies, including those on COVID-19. Body fluid proteomics is considered an emerging technology for identifying novel biomarkers because they are informative clinical samples of infectious diseases. This review also introduces recent MS analyses for body fluid proteomics: DDA, DIA, and targeted-mass spectrometry (PRM and MRM). In general, pathogenic proteins or peptides have extremely low copy numbers of bodily fluids. Therefore, the authors suggest that further innovations in MS instruments and informatics are required for practical applications.

The metaproteome is defined as ‘all proteins identified and quantified from complex microbial communities’. This term is derived from the metagenome. Normally, we can obtain valuable information on the major metabolic enzymes of specific bacterial communities by metaproteomic analysis. Zhang et al. [6] applied this approach to discover highly active urease in the rumen microbiota of cattle. Urease in ruminant animals is important because it plays a crucial role in regulating nitrogen emissions to the environment. Optimized protein extraction and purification methods for the urease active fraction were designed and gel-based LC-MS/MS analysis was performed. Finally, they identified six active microbial ureases from 2225 rumen proteins. These active ureases could be targets for designing novel urease inhibitors to regulate rumen microbial urease activity.

Charteau et al. [7] applied a label-free proteomic assay to understand the functions of major cytochromes of *Bacillus cereus*. Proteomic analysis of the deletion mutant (∆*ctaA*) revealed altered proteome remodelling in *Bacillus cereus* to compensate for the loss of cytochrome *caa3* activity. This also explains how the deletion mutant strain is more resistant to cold and oxidative stress at the proteomic level. *Bacillus thuringiensis* is a pathogen with highly effective insecticidal properties. The categorization of these strains was based on a serotyping assay using flagellins as reference proteins. However, Shikov et al. [8] proved that this established serotyping method was not supported by phylogenic analysis using genomic properties. Comparative genomic and proteomic techniques were used to draw conclusions.
